# Aberrant Free Radical Biology Is a Unifying Theme in the Etiology and Pathogenesis of Major Human Diseases

**DOI:** 10.3390/ijms14048491

**Published:** 2013-04-17

**Authors:** Frederick E. Domann

**Affiliations:** Departments of Radiation Oncology, Surgery, and Pathology; Carver College of Medicine, the University of Iowa, Iowa City, IA 52242, USA; E-Mail: frederick-domann@uiowa.edu; Tel.: +1-319-335-8018; Fax: +1-319-335-8039

**Keywords:** mitochondria, oxidative stress, reactive oxygen, nitric oxide, free radicals, cancer, diabetes, vascular disease, neurodegenerative disease, aging

## Abstract

The seemingly disparate areas of oxygen toxicity, radiation exposure, and aging are now recognized to share a common feature—the aberrant production and/or removal of biologically derived free radicals and other reactive oxygen and nitrogen species (ROS/RNS). Advances in our understanding of the effects of free radicals in biology and medicine have been, and continue to be, actively translated into clinically tractable diagnostic and therapeutic applications. This issue is dedicated to recent advances, both basic discoveries and clinical applications, in the field of free radicals in biology and medicine. As more is understood about the proximal biological targets of aberrantly produced or removed reactive species, their sensors, and effectors of compensatory response, a great deal more will be learned about the commonalities in mechanisms underlying seemingly disparate disease states. Together with this deeper understanding, opportunities will arise to devise rational therapeutic interventions to decrease the incidence and severity of these diseases and positively impact the human healthspan.

Aberrant production and/or removal of biologically derived free radicals and other reactive oxygen and nitrogen species (ROS/RNS) is a common feature of many human diseases, as well as radiation exposure, oxygen toxicity. Advances in our understanding of the effects of free radicals in biology and medicine have been, and continue to be, actively translated into clinically tractable diagnostic and therapeutic applications. This issue is dedicated to recent advances, both basic discoveries and clinical applications, in the field of free radicals in biology and medicine.

Illustrative of the breadth and depth of the now mature field of free radical biology and medicine, this special issue (www.mdpi.com/journal/ijms/special_issues/free_radicals) contains 24 rigorously peer-reviewed manuscripts representing authors from 11 different countries (Brazil, Canada, China, Italy, Korea, Mexico, Netherlands, Slovenia, Spain, Taiwan, and United States) across four continents (Asia, Europe, North America and South America) spanning the globe. Not only is interest in free radical biology and medicine broad geographically, but also across a broad spectrum of medical specialties that include the diagnosis and treatment of many or most of the major diseases and disorders of mankind. As a result, we believe the issue is well balanced, consisting of 14 original research articles and 10 review papers covering a range of topics encompassing the role(s) of free radicals and ROS or nitric oxide in the etiologies and/or the molecular pathologies of nearly all of the major human diseases including cancer [1,2], vascular diseases [3–5], diabetes [6], and neurodegenerative disease [7]. One cancer study reported results from an analysis of mitochondrial adaptations in lymphoma [2], while the other associated a high mitochondrial DNA copy number with increased invasiveness in esophageal squamous cell cancer [1]. The vascular biology papers included reports regarding both cerebral vascular [4] and pulmonary vascular [3] endothelial cell function. Also included in this issue are papers describing the contributions of ROS and mitochondrial function to other more rare and specific disease states including lupus [8] and epilepsy [9] and chronic regional pain syndrome [10]. Popular topics in this special issue include mitochondrial function [1,2,5,8,11], oxidative stress [2,6,12–15], nitric oxide [3,16–18], cellular signaling [3,13,19]. In addition there are reports pertaining to endoplasmic reticulum stress [12], hypoxic stem cell niches [20], adaptive responses to oxidative stress [21], and a paper describing a Columbian medicinal plant that inhibits lipid peroxidation [22]. There are papers relating to the role of ROS and nitric oxide in different organ systems including brain [7,15], muscle [11,17], as well as papers detailing the role of reactive oxygen and free radical biology in mediating the toxicity of ischemia [13]. Finally, there are three papers that present significant technical advances; two on the *in vivo* detection of ROS [15,23], and one that introduces an advanced Electron Spin Resonance technique to study lipid peroxidation [24]. The word-cloud in Figure 1 is a convenient visual representation illustrative of the frequency of use of particular words in the titles of the 23 manuscripts contained in this special issue.

It is hoped that this special issue will spark discussion across disciplines about the fundamental role that free radicals and ROS/RNS play in the fundamental underlying causes to many of the major diseases of mankind. As more is learned about the proximal biological targets of aberrantly produced reactive species, their sensors, and effectors of compensatory response, it is likely that even more commonalities in mechanism(s) underlying seemingly disparate disease states will emerge. Together with this deeper understanding, opportunities will arise to devise rational therapeutic interventions to decrease the incidence and severity of these diseases and positively impact the human healthspan.

## Figures and Tables

**Figure 1:**
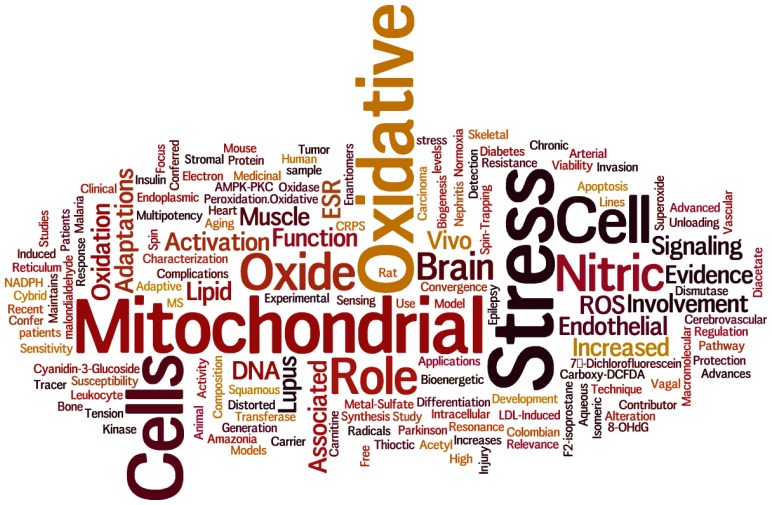
A word cloud created using the titles of all 24 peer-reviewed articles in this special issue. The relative size of each word is weighted according to the frequency with which it is used among all titles.
